# Attempted suicide and suicide of young Turkish women in Europe and Turkey: A systematic literature review of characteristics and precipitating factors

**DOI:** 10.1371/journal.pone.0253274

**Published:** 2021-08-04

**Authors:** Diana van Bergen, Ozlem Eylem-Van Bergeijk, Amanda Heredia Montesinos

**Affiliations:** 1 Department of Pedagogics and Education, Faculty of Social and Behavioural Sciences, Groningen University, Groningen, The Netherlands; 2 Department of Clinical Psychology, VU University, Amsterdam, The Netherlands; 3 Wolfson Institute of Preventive Medicine, Queen Mary University of London, London, United Kingdom; 4 Psychiatric University Clinic of Charité at St. Hedwig Hospital, Berlin, Germany; Ege University, School of Medicine, TURKEY

## Abstract

**Background:**

The increased risk of suicidal behaviour among Turkish women living in Europe and Turkey is a serious public health problem. This study compares and synthesises the empirical evidence of demographic, social, psychological and interpersonal characteristics and precipitating factors in the suicides and attempted suicides of Turkish women in Europe and Turkey.

**Methods:**

We systematically searched eight databases (PsycINFO, PubMed, Med Line, Web of Science, Smart Cat, Safety Lit, BASE and Ulakbim), using search terms in English, Turkish, German and Dutch, as well as the reference lists of the retrieved papers. We extracted data on countries/regions, population characteristics, sample characteristics, recruitment, method of data collection, type of suicidal behaviour (suicide or attempted suicide) and precipitating factors and characteristics. The results were qualitatively synthesised.

**Results:**

We retrieved nine studies on attempted suicide in Europe (from Germany, Switzerland and the Netherlands), 17 studies on attempted suicide in Turkey and 10 studies on suicide in Turkey (36 in total). Overall, we found similar precipitating factors and characteristics of attempted suicide and suicide in Turkey and Europe, including socio-demographic factors (young age and not being enrolled in the labour market), poverty and, to some extent, mental illness. Moreover, conflicts with family or spouses and violence against women, including so-called honour violence, were particularly common for women living in or originating from traditional areas in Turkey.

**Conclusion:**

The framework of intersectionality is relevant to understanding our results, because structural inequalities in gender roles, gender role expectations as well as power imbalances among socio-economic classes collectively impact the suicidal behaviour of Turkish women. Moreover, the importance of violence against women points to the cultural continuity of the patriarchal and oppressive structures of Europe and Turkey. Suicide prevention efforts should address cultural attitudes underlying violence against women and girls through community education programmes, cultural and gender-sensitive care provision and jurisdiction.

## Background

Suicide and attempted suicide affect millions of people worldwide and are major tragedies at the family, community and personal level. Suicide refers to “death resulting from intentional self-injurious behavior, associated with any intent to die as a result of the behavior”, while *attempted* suicide refers to “a potentially self-injurious act committed with at least some intent to die, as a result of the act.” [1] Globally, it is well documented that in most countries, women attempt suicide more often than men, although men die by suicide more often than women, partially because they use more lethal methods [2, 3]. An exception to this longstanding gender pattern in suicide deaths is the high number of women who die by suicide in certain Muslim-majority countries in the Middle East and parts of Asia [2, 4–8]. This variation is even more striking when stratified by rural (generally more traditional and somewhat more religious) regions versus urban (generally less traditional and somewhat more secular) regions. For instance, studies have suggested that young Pakistani women living in rural and/or remote areas [6], women in rural Iran [7] and Turkish women and girls in rural areas of Eastern Turkey [8, 9] have a higher risk of suicide than males in these regions. Therefore, we choose one of these Muslim/majority female groups in relation to suicidal behaviour, that is, Turkish females, as the topic for investigation for this paper. For Turkish females, studies have shown a similarly disproportionate risk of suicidal behaviour among those who have migrated to European countries, such as Germany, Switzerland or the Netherlands [10]. This is why the suicidal behaviours of Turkish females who live in Turkey as well as Turkish female immigrants to Europe need further investigation [11].

The epidemiology of suicidal behaviour of Turkish women (residing in Turkey) shows that the suicide rate among Turkish women aged 15–24 in Eastern Turkey is twice as high as their male counterparts since 2000 [9]. These finding were recently replicated by an annual report on suicide statistics drawn from Turkey’s general population [12]. Similarly, hospital registration data in Southeast Turkey has shown that attempted suicide rates were 4.15 times higher among young women 15–24 years old who presented at emergency departments compared to men of the same age as well as older men and women [11, 13].

Focussing on the epidemiology of suicidal behaviour of Turkish female immigrants in Europe, hospital registration studies show that in the Netherlands [14], Germany [15] and Switzerland [16], the risk of attempted suicide was at least 2.5 times higher among Turkish immigrant women aged 14–25 who presented at emergency departments compared to Turkish immigrant men of the same age and women in the host country. Furthermore, 10–17-year-old girls and young women of Turkish descent had an almost twofold risk of suicide compared to ethnic majority women in Germany (relative risk = 1.79; 95% CI = 1.41–2.27) [17].

Regarding the demography of migrants in Europe, it is striking that immigrants from Turkey are among the largest ethnic minority populations in multiple western European countries. Most came as contracted foreign labourers in the 1960s and 1970s from predominantly rural and traditional areas in Turkey [18]. The countries that received the most Turkish immigrants were Germany, Switzerland, Austria and the Netherlands, which have Turkish immigrant populations of 5%, 5%, 3% and 3%, respectively [18]. People who migrate from Turkey to Europe are predominantly Muslim (approximately 95%), and most are Sunni [18]. During the 1960s and 1970s, socio-economic deprivation, especially in the rural areas of Eastern Turkey, pushed migrants to seek employment in Western Europe, which eventually led to family reunification and cross-national marriages. More recently, migration from Turkey has been triggered by political instabilities [19, 20].

The elevated risk of suicidal behaviours among young Turkish women in Turkey and female Turkish immigrants in Europe raises important questions that we will address in this review: 1) what characteristics and precipitating factors contribute to the elevated risk of suicidal behaviour among Turkish females? 2) To what extent is there similarity in the characteristics and precipitating factors contributing to suicidal behaviour of Turkish women residing in Turkey versus in Europe? We use the term ‘precipitating factors’ to describe perceived causes or contributors to the manifestation of suicidal behaviour, and these perceptions can be either participants’ views or researchers views (depending on the methodology used in studies).

## Precipitants and characteristics of suicidal behaviours among Turkish women

Mental illness is often listed as a critical factor in suicidal behaviours in individuals [21]. In a study from Southern Turkey, depression was three times more likely to be reported among women than men who attempted suicide (72% versus 27%; [22]). Similarly, depression was more often reported among Turkish female immigrants who attempted suicide in Germany compared to German majority women (51% versus 34%; [23]). Although depression and other mental illnesses have been linked to suicide, several authors have argued that they are not the ultimate cause of suicidal behaviour among women of certain cultural backgrounds. Indeed, these authors suggested that mental illness and suicidal behaviours among women in the Middle East are at least partially a consequence of aggression or violence against women rather than an independent factor or psychopathology [2]. A similar argument has been put forward for immigrant women from Turkey and South Asia in Europe who have demonstrated suicidal behaviour [24, 25].

Turkey’s socio-cultural landscape is characterised by a variety of ethnicities, and religious convictions and relatedly, a diversity of values and norms. Ethnic Turks and Kurds together comprise the largest ethnic groups in Turkey, but the country also hosts ethnic populations much smaller in size, for instance Circassian, Bosnian, Albanian, Georgian and Arab Turks [19, 20]. Most Turks are Sunni Muslims (81%), but a minority are Alevis, a group that has a more secular lifestyle than Sunnis [18]. Traditionalism (e.g., strong identification with Islamic ideology and a patriarchal family life) is common in the central and eastern regions, while secular lifestyles and egalitarian views of gender roles are more common in the western regions [26]. The concepts of *namus* and *seref* (i.e., honour) are central to how gender roles are expressed according to the patriarchal system of rural Turkey [10, 26]. *Namus* holds women responsible for their family’s honour by having them maintain their sexual abstinence until they get married and requiring them to demonstrate modesty in their interactions with the opposite sex. Failing to do so can result in the loss of a family’s reputation [26]. In extreme cases, so-called honour killings are committed by male family members and/or male community members against women who are seen to jeopardise their family’s reputation [26, 27].

*Namus* and *seref* can play a role in the manifestation of suicidal behaviours by women living communities in traditional rural Turkey [9, 28, 29]. There is also evidence that these phenomena can be transported to Europe upon migration by Turkish people. Studies showed that honour-related violence and aggression were relevant to suicidal behaviours among young Turkish immigrant women in Germany [10], the Netherlands [24]. Honour-related violence was also reported among young women in other Muslim-majority countries in the Middle East [6–10, 30]. Furthermore, empirical findings of the impacts of honour-related violence for suicidal behaviour resonate with Durkheim’s archetype of *fatalistic suicide* [24, 31, 32]. According to this archetype, suicide is a reaction to the harsh moral demands of the community, which are upheld by force [24]. When strict moral rules are experienced by women as external, limiting, demanding and obtrusive, they may experience hopelessness and, ultimately, suicidal thoughts [8, 31, 32]. The consistency of honour-related violence among Turkish women in Europe and Turkey [24, 25] might be indicative of the continuity of Durkheim’s fatalistic suicide after migration.

Besides the fatalistic suicide model, explanations of the elevated rates of suicidal behaviours among female Turkish immigrants in Europe include difficulties in the migration and acculturation processes [25, 27]. Several studies report that ‘culture conflicts’ cause decreased psychological well-being and suicidality among female Turkish immigrants in Europe [33, 34]. Studies of culture conflicts suggest immigrant women may perceive an unbridgeable gulf between the need to fulfil modern social roles in mainstream society and their abiding the collectivism and traditionalism required by their families and communities [10, 24, 33]. These issues have an impact on suicidal behaviour [24, 25].

Furthermore, socio-economic factors are known to play a role in the mental health and wellbeing of immigrant populations. Turkish immigrants in Europe often suffer from a disadvantaged socio-economic position compared to the majority of the population, including a lack of adequate housing, optimal schooling and access to culturally sensitive mental healthcare. Socio-economic disadvantages are known to predict distress, depression and, ultimately, suicidal behaviour and are therefore relevant to understanding suicidality among Turkish immigrant women [35]. In summary, the intersection of socio-economic factors, honour-related violence, mental illness and culture conflicts is crucial to the understanding of suicidal behaviour among Turkish women [30, 36].

## The present study

The heightened risk of suicidal behaviours among Turkish women living in Turkey and Europe is a serious public health problem, and a comprehensive systematic review of this issue is currently lacking. Thus, in this study, we compared and synthesised the empirical evidence of the characteristics and precipitating factors in the suicides and attempted suicides of Turkish women in Turkey and Europe. Specifically, we investigated the relevance of 1) socio-demographic; 2) migration-related (e.g., acculturative stress) and, 3) gender-related factors (e.g., honour-related violence and other forms of violence against women). We choose these three categories as previous research among women and immigrants has shown the relevance of these categories to the emergence of their suicidal behaviour [21, 25, 27]. We also examined the role of psychiatric illness, although we are aware that it might be caused by the aforementioned factors. Since rates of suicidal behaviours and the accompanying precipitating factors may vary depending on degree of traditionalism of the area, we classified the research sites into ‘traditional areas’ and ‘less traditional areas’ (these were assessed by OE and checked by a sociologist from Turkey). We use the term ‘precipitating factors’ when referring to the perceived causes of or important contributors to suicidal behaviour (e.g., different forms of violence) and ‘characteristics’ when referring to the aspects that increase the likelihood of experiencing a suicidal crisis (e.g., psychiatric illness) and/or are not necessarily causal [2, 36]. Some studies distinguished between first-generation versus second-generation immigrant women, terms that respectively refer to whether an individual was born in Turkey and migrated to a host country or was the child of immigrants who moved to the host country.

## Methods

### Selection and identification of the studies

#### Search strategy

We searched the following bibliographic databases: PsycINFO, PubMed, Med Line, Web of Science, Smart Cat, Safety Lit, BASE and Ulakbim. The Turkish database Ulakbim was chosen because it is a comprehensive database that includes articles in Turkish from several Turkish databases, such as Tübitak (i.e. a comprehensive database of scientific publications from variety of disciplines in Turkish, see https://ulakbim.tubitak.gov.tr/en) and Sosyal Bilimler (i.e. a comprehensive database of scientific publications from social sciences such sociology, history, anthropology etc., see https://www.sosyalbilimler.org/). We also checked the reference lists of all the papers we included in our last step in order to identify additional studies [37]. The following terms were used as index terms or free-text words (including synonyms and closely related words): ‘suicide’, ‘deliberate self-harm’ and ‘Turk’. Additional language filters were used to search Turkish, German and Dutch articles within our retrieved records. There is no review protocol for this study. The string for the electronic search strategy was as follows:

1. ‘suicide’
2. ‘deliberate self-harm’
3. 1 or 2
4. ‘Turk’
5. 3 and 4

#### Inclusion criteria

The inclusion criteria were 1) articles presenting original empirical data on demographic, psychological, psychiatric or social factors relevant to the suicide or attempted suicide among Turkish girls and women living in Turkey and Europe; 2) articles using hospital samples, crisis-centre samples (for attempted suicide) and/or forensic reports (for suicide); and 3) articles published between 1960 and 2020.

#### Exclusion criteria

The exclusion criteria were 1) articles that investigated suicidal ideation only, 2) articles that did not investigate gender differences, 3) articles that only investigated geriatric populations, and 4) articles that only reported standardised mortality rates, prevalence or incidence data.

#### Quality assessment

In line with a multidisciplinary approach to examining the characteristics and precipitants of suicidal behaviours [21], multiple research designs were included in the review. The following core criteria were used to analyse the studies (see Table 1): 1) whether specific information about women was assessed and provided (beyond speculation in the Discussion), 2) whether precipitating factors and characteristics of suicide and/or attempted suicide were assessed and reported, 3) whether the articles was published in a peer-reviewed journal, 4) whether the research questions and study design were clear, 5) whether there was a comparison group from the majority ethnic group and/or healthy controls in case-control studies, 6) whether the information on attempted suicide was collected through a retrospective analysis. 7) whether the researchers directly gathered information from the patients and/or relatives (through self-report questionnaires and/or interviews) and 8) whether there was a follow-up assessment.

**Table 1:** Quality assessment of the studies.

Questions: 1) Whether specific information on women was assessed and provided (beyond merely a speculation in the discussion)								
2) Whether precipitating factors and characteristics of suicide and/or attempted suicide were measured and reported								
3) Whether the study was published in a peer reviewed journal								
4) Whether research questions and design of the study were clear								
5) In the case of a case-control study: Whether there was a comparison group from the majority ethnic group and/or healthy controls								
6) Whether the information was collected retrospectively only (through the evaluation of the case reports)								
7) Whether the information was gathered from those who attempted and/or completed suicide, and/or relatives by the researchers (through interviews and/or self-report measures)								
8) Whether there was any follow-up assessment to re-assess characteristics and/or precipitating factors of attempted suicide								
Study	1	2	3	4	5	6	7	8
Aichberger et al. (2015) [23]	**✓**	**✓**	**✓**	**✓**	X	X	**✓**	X
Brückner et al. (2011) [38]	**✓**	✓*	**✓**	**✓**	X	**✓**	X	X
Burger et al. (2010) [14]	**✓**	✓*	**✓**	**✓**	X	**✓**	X	X
Burger et al. (2015) [39]	**✓**	✓*	**✓**	**✓**	X	**✓**	X	X
Heredia Montesinos et al. (2019) [25]	**✓**	**✓**	**✓**	**✓**	X	X	**✓**	X
Özlü-Erkilic [40]	**✓**	✓*	**✓**	**✓**	X	**✓**	X	X
Van Bergen et al. (2009) [24]	**✓**	**✓**	**✓**	**✓**	X	**✓**	X	X
Yilmaz and Riecher-Rossler (2008, 2012) [41, 42]	**✓**	**✓**	**✓**	**✓**	**✓**	X	**✓**	X
Akın, Tüzün & Çil (2007) [43]	**✓**	**✓**	**✓**	**✓**	X	X	**✓**	X
Cetin et al. (2001) [44]	**✓**	**✓**	**✓**	**✓**	X	X	**✓**	X
Devrimci et al. (2003) [11]	**✓**	**✓**	**✓**	**✓**	X	**✓**	X	X
Eroglu et al. (2013) [45]	**✓**	**✓**	**✓**	**✓**	X	X	**✓**	X
Erşan & Kılıç (2013) [46]	**✓**	**✓**	**✓**	**✓**	X	**✓**	X	X
Guloglu et al. (2009) [47]	**✓**	✓*	**✓**	**✓**	X	**✓**	X	X
Ibiloglu et al. (2016) [48]	**✓**	✓*	**✓**	**✓**	X	X	**✓**	X
Konkan et al. (2014) [49]	**✓**	**✓**	**✓**	**✓**	**✓**	X	**✓**	X
Köse et al. (2012) [50]	**✓**	**✓**	**✓**	**✓**	X	**✓**	X	X
Mete et al., (2019) [51]	**✓**	**✓**	**✓**	**✓**	X	**✓**	X	X
Ozdel et al. (2009) [52]	**✓**	**✓**	**✓**	**✓**	X	**✓**	X	X
Saraçoğlu et al. (2014) [22]	**✓**	✓*	**✓**	**✓**	X	X	**✓**	X
Senol et al. (2005) [53]	**✓**	**✓**	**✓**	**✓**	X	X	**✓**	X
Simsek et al. (2013) [13]	**✓**	**✓**	**✓**	**✓**	X	**✓**	X	X
Turhan et al. (2011) [54]	**✓**	**✓**	**✓**	**✓**	X	**✓**	X	X
Yasan et al. (2008) [55]	**✓**	**✓**	**✓**	**✓**	X	X	**✓**	**✓**
Yektaş et al. (2014) [56]	**✓**	**✓**	**✓**	**✓**	X	X	**✓**	X
Altindag et al. (2005) [28]	**✓**	**✓**	**✓**	**✓**	X	**✓**	X	NA
Asirdizer et al. (2010) [57]	**✓**	**✓**	**✓**	**✓**	X	**✓**	X	NA
Enginyurt et al. (2014) [58]	**✓**	**✓**	**✓**	**✓**	X	**✓**	X	NA
Goren et al. (2004) [59]	**✓**	**✓**	**✓**	**✓**	X	**✓**	X	NA
Hekimoglu et al. (2016) [29]	**✓**	**✓**	**✓**	**✓**	X	**✓**	X	NA
Karbeyaz et al. (2013) [60]	**✓**	**✓**	**✓**	**✓**	X	**✓**	X	NA
Karberyaz et al. (2016) [61]	**✓**	✓*	**✓**	**✓**	X	**✓**	X	NA
Oner et al. (2014) [12]	**✓**	**✓**	**✓**	**✓**	X	**✓**	X	NA
Oner et al. (2007) [62]	**✓**	**✓**	**✓**	**✓**	X	**✓**	X	NA
Taktak et al. (2013) [63]	**✓**	✓*	**✓**	**✓**	X	**✓**	X	NA

✓: Yes; X: No;

*: Social precipitants (i.e. relationship and family problems, domestic violence and sexual abuse, ‘honour’-related violence and/or others) were not investigated and/or reported; NA: Not Applicable (follow-up assessment of precipitants and characteristics of suicide attempt is not applicable to the studies investigating suicide).

**Fig 1:**
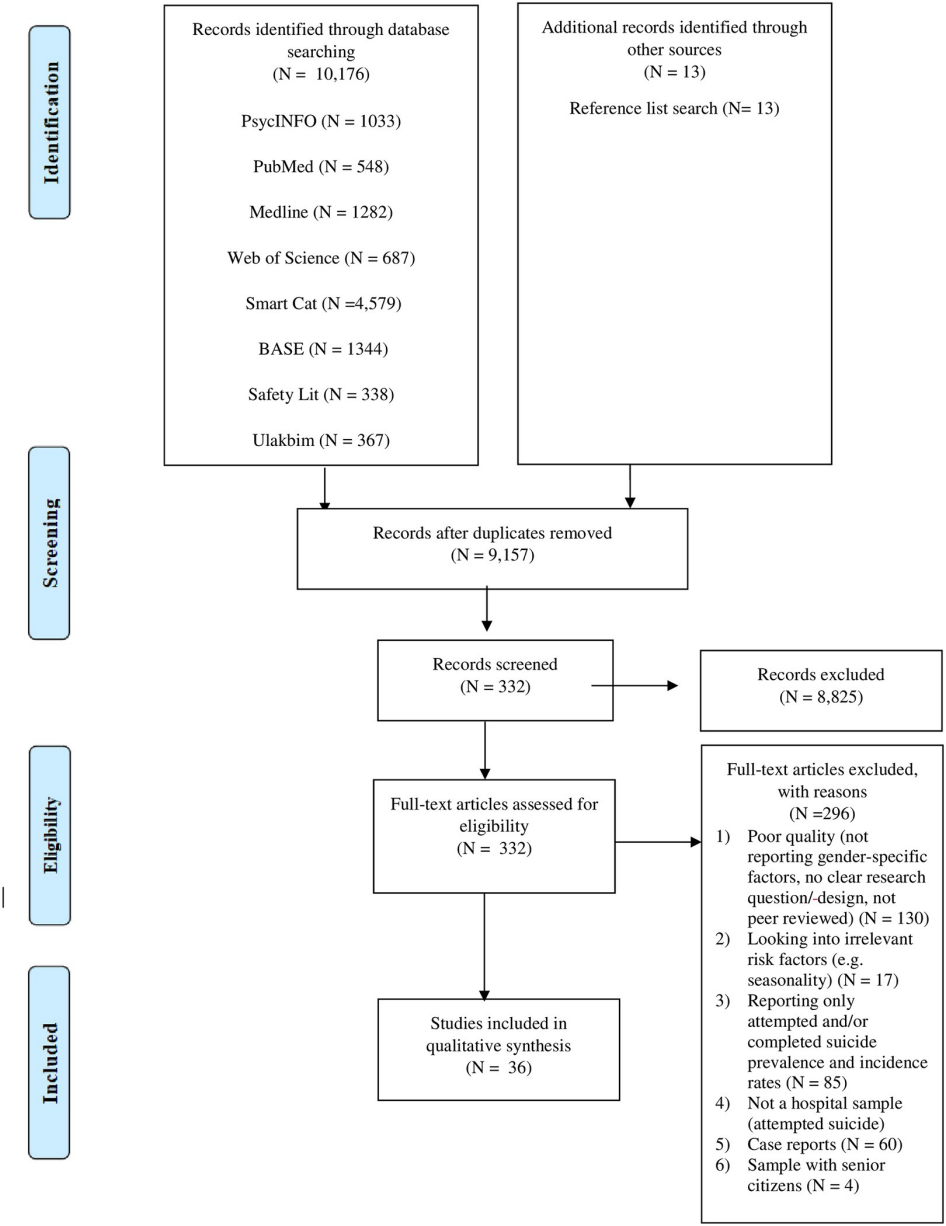
Prisma flowchart: Flow of studies on suicide and attempted of Turkish women in Turkey and Europe. Eligible studies were retrieved by the systematic literature search and article selection process, consisting of the following steps: identification of records, screening for inclusion, assessing of studies eligibility, and inclusion of studies into the integrative research review.

Furthermore, the quality of autopsy studies was evaluated by consulting the guidelines for such study designs [64]. The studies that did not meet the first four criteria were excluded (see Fig 1). Two authors (DvB and OE) were involved in the quality assessment, and the discrepancies between the authors’ selections were resolved through discussions.

#### Data extraction

A customised data extraction form was generated in consultation with the Cochrane Collaborative GRADE approach [37]. This form included but was not limited to countries/regions, population characteristics, sample, place and year of recruitment, methods of data collection, type of suicidal behaviour (suicide or attempted suicide), socio-economic factors, age, gender, precipitating factors, social stressors and psychiatric factors. The titles of all articles were screened, and the abstracts of the articles were checked regarding our inclusion criteria. When no definitive decision could be made based on the abstract, the original articles was reviewed. Two reviewers (DvB and OE) independently completed the extraction form, and a third reviewer (AHM) established consensus across the two forms. After the selection process, we retrieved the full texts of the articles that met the criteria in order to perform our analyses.

## Results

After examining a total of 10,189 abstracts, we retrieved 332 full-text papers for further consideration. We excluded 9,857 retrieved papers. A PRISMA flowchart [37] describing the inclusion process is presented in Fig 1. A total of 36 studies were included in the qualitative synthesis; results of the characteristics and precipitating factors are summarised in Table 2.

**Table 2:** Characteristics and precipitating factors of attempted suicide and suicide of Turkish women in Turkey and Europe.

Authors	Country, Region	Type of Suicidal Behaviour	Sample	Place and year of Recruitment	Methods	Findings
Aichberger et al. (2015) [23]	Berlin, Germany	Attempted suicide	N = 159 Turkish female migrants	10/2008-09/2011 40 Emergency departments, 3 psychiatric clinics	Surveillance study. Data collection by emergency unit staff, and psychiatrists. Standardized documentation form (socio-demographics, clinical data, and reasons for the attempt).	**Characteristics** Mental Illnesses Psychiatric disorders (78,6%) Adjustment disorder (49.7%) Depression (22.0%) Psychotic or personality disorders (13.8%) **Precipitating Factors** Prior conflict with their spouse or own family (first generation: 61%, second generation: 34.6%) Conflict with parents (more common in second generation) Mental illness (13.8%) Work-related problems (10.7%) Difficulties with coping with physical illness (1.2%), Domestic violence (0.6%) Financial strains (0.6%)
Brückner et al. (2011) [38]	Basel, Switzerland	Attempted suicide	N = 56 Turkish immigrants N = 36 females N = 291 Native Swiss N = 210 females	2003–2004 Hospitals in Basel Kanton that participated in the WHO/ EURO -Multicentre study	Surveillance study. Data collection by hospital staff. Standardized anonymized documentation form (clinical and socio-demographic data).	**Characteristics** Socio-demographic factors • Young age (Turkish women between age 15–24 & 35–39) (Mental) Illness Affective disorder (51.4%) Stress-related somatoform disorders (45.7%) were more common compared to Turkish men (40%, & 25%) and Swiss females (40.6% & 30,6%) Personality disorders (2.9% vs 10%) were more common compared to Turkish men & Swiss females (2.9% vs 35.4%)
Burger et al. (2010) [14]	The Hague, The Netherlands	Attempted suicide	N = 1458 suicide attempts N = 128 suicide cases	2002–2004 Hospitals located in The Hague and its suburbs	Surveillance study. Registration form filled out by hospital staff. Staff asked questions to patients about demographics and reasons for attempt.	**Characteristics** Socio-demographic factors Young age Turkish females aged 15–25 (575/100.000 vs. 310/100.000 in the Dutch group) No difference in socioeconomic living conditions compared to Dutch women
Burger et al. (2015) [39]	The Hague, The Netherlands	Attempted suicide	N = 1534 N = 842 Dutch N = 209 Surinamese N = 137 Turkish N = 25 Antillean	2008–2010 Hospitals located in The Hague and its suburbs	Surveillance study Registration form filled out by hospital staff, who asked patients about demographics, diagnoses reasons for attempt.	**Characteristics** (Mental) Illness • Mental illnesses were less common (except depression) compared to Dutch women (7% vs. 36%)
Heredia Montesinos et al. (2019) [25]	Berlin, Germany	Attempted suicide	N = 15 attempters, N = 20 women from Turkish community	2011–2015 Recruited through psychiatric clinics, practitioners, psychiatrists, psychotherapists, and women´s shelters	Focus groups and questionnaire (socio-demographics)	**Characteristics** Socio-demographic factors Level of high education among first generation of suicide attempters (0%), second generation of suicide attempters (20%) Unemployed (40%) in first generation and (30%) in second generation (Mental) Illness Affective disorders (73.3%) Neurotic/stress-related somatoform disorder (20%) **Precipitating Factors** Impact of family and community: pressures due to collectivism (family-centeredness leading to neglect of self-care and autonomy) and due to family honour in Turkish culture The impact of German society: racism, discrimination, and lack of acceptance
Özlü-Erkilic (2019 [40]	Vienna, Austria	Attempted suicide	N = 1718 patients were aged between 4–18 years N = 918 (53.4%) had a migration background N = 800 (46.6%) were native Austrians N = 130 (7.6%) patients had parents with a migration history from Turkey.	2011–2014 Emergency Unit of the Department of Child and Adolescent Psychiatry at the Medical University of Vienna.	Retrospective evaluation of the hospital records Hospital staff administered monitoring forms of patients treated for attempted suicide	**Characteristics** Socio-demographic factors: Ethnicity: Turkish-speaking young people were at more than 2 times (OR = 2.21, 95% CI: 1.408–3.477, p = 0.001) higher risk for suicide attempts, as compared to other patient groups. Gender: In the Turkish speaking group, three quarters (71.5%) of them being females **Precipitating factors** • Attempted suicide among the Turkish speaking youth was triggered almost 3 times (OR = 2.94, 95%CI: 1.632–5.304) more often by interfamilial conflicts compared to natives, and this concerned females more often than males*
Van Bergen et al. (2009) [24]	Amsterdam, The Netherlands	Attempted suicide	N = 115 (female only sample aged 12–40)	1996–2005 Hospital record and/or case files from VU University Hospital Emergency Unit and a Crisis Centre that mentioned a suicide attempt were included	Retrospective evaluation of the hospital crisis centre records. Medical case files filled out by hospital staff or crisis centre staff. Clinical interviews were held with women and reasons for attempt were asked for. Sometimes medical expert reports were added to the case file.	**Characteristics** (Mental) Illness Psychiatric or personality disorder (Dutch 79%, Turkish 59%, Moroccan 60%, Suriname 71%) Co-morbidity (Dutch 59%, Suriname 33%, Turkish 28%, Moroccan 27%) **Precipitating Factors** In at least half of the cases, stressful life events related to family honour and personal autonomy restriction existed. Physical abuse (Turkish 31%, Dutch 21%) Sexual abuse (Turkish 19%, Dutch 38%) Overregulation: 55% of cases for Turkish vs. Dutch: 28% Demand for maintaining sexual abstinence (30% Turkish) Fear of being an outcast (Turkish 9%, Dutch 0%) Being forced to maintain unwanted marriage (Turkish 6%., Dutch 0%) Rejection of partner (Turkish 9%, Dutch 3%,)
Yilmaz and Riecher-Rossler (2008) [41]	Basel, Switzerland	Attempted suicide	N = 70 Turkish	1991–1997 Emergency Unit of University Hospital of Basel-City	Surveillance study. Data collection through medical files and evaluation form filled out by attending physician (socio-demographics, main reasons for the attempts, diagnoses).	**Characteristics** Socio-demographic factors First generation: 56% females Second generation: 75.6% females **Precipitating Factors** Relationship problems: first generation: 60%, second Generation: 64.4% Problems with parents: first generation: 0%, second generation: 20% Threat of deportation: first generation: 16%, second generation 4.4% Domestic violence within family and partnership (24.1% first. generation, 14.7% second generation)
Yilmaz and Riecher-Rossler (2012) [42]	Basel, Switzerland	Attempted suicide	N = 271 N = 46 Turkish N = 225 Swiss	2003–2004 Emergency Unit of University Hospital of Basel-City WHO–EURO Multicentre Study on Suicidal Behaviour	Surveillance study. Data collection through hospital staff. Standardized anonymized documentation form (clinical and socio-demographic data).	**Characteristics** Socio-demographic factors Suicide attempt rates among Turkish immigrants 2.7 times higher than among Swiss Turkish: 36% males, 64% females Swiss: 28% males, 72% females Highest rates among Turkish females aged 15–24 and 35–39 (Mental) illness Affective disorders: 51.4 Turkish females vs. 30.6% Swiss females Adjustment disorder: 45.7% Turkish females vs. 14.6% Swiss females
Akın, Tüzün & Çil (2007) [43]	Diyarbakir, South-East Turkey, traditional	Attempted suicide	N = 80 N = 55 females N = 25 males	2005–2006 Diçle University Hospital	Clinical interviews with the patients admitted to the hospital	**Characteristics** Socio-demographic factors Female gender (69% of attempts by females) Young age 57% in15-25 age group (vs 35%: 25 and older according to the Turkish Ministry of Statistics) **Precipitating Factors** Domestic violence (24%) Being unemployed/economically dependent on their husbands*
Cetin et al. (2001) [44]	Ankara, Central Anatolia, less traditional	Attempted suicide	N = 33, N = 23 females, N = 10 males (youth) Attempters, did not obtain mental health treatment N = 50 (N = 33 females, N = 17 males) controls who received mental health treatment N = 50 controls (N = 26 females, N = 24 males)	Data collection period was not specified Child, Adolescent and Adult Psychiatry Departments of Hacettepe University Medical Center	Case-control study Demographic information and self-reported data from patients on their mental health were obtained through validated scales.	**Characteristics** (Mental) Illness • Depression was more common in female psychiatric outpatients (who had not attempted) than males in the other two groups* **Precipitating Factors** Negativity in the familial aspect of the self-image (i.e. problems with the family was more often reported by females. Negative self-image in the family was more often reported by females and seen as an important factor separating suicide attempters from non-attempters)*
Devrimci-et al. (2003) [11]	Mamak- Ankara, Central Anatolia, less traditional	Attempted suicide	N = 737 N = 514 females N = 223 males	1998–2001 5 hospitals & 22 primary care units WHO–EURO Multicentre Study on Suicidal Behaviour	Retrospective evaluation of the hospital records. Hospital staff administered monitoring forms of patients treated for attempted suicide	**Characteristics** Socio-demographic factors Female gender (69.74% of attempts by females vs. 30.26% by males) Young age (15–24) (1999: females 64.93% vs 35.07%; 2000: females 69.32% vs 30.60%; 2001: females 70.53% vs 29.47% males) Unemployment (68% females vs 32% males) Not participating in labour force (57% females were mostly housewives and college students)
Eroglu et al. (2013) [45]	Erzurum & Erzincan province, North Eastern Anatolia, traditional	Attempted suicide	N = 893 N = 660 females N = 230 males	2006–2008 17 emergency rooms of state hospitals	Clinical interviews with the patients admitted to the hospital	**Characteristics** Socio-demographic factors Female gender (female to male ratio: 3:1)* Young age (age group 15–24 had a female to male ratio: 3.6:1)* Being married (42% females vs. 31% males) Unemployment (64% females vs. 31% males) **Precipitating Factors** Family problem (30% females vs. 21% males) Domestic violence (9% females vs. 2% males)
Erşan & Kılıç (2013) [46]	Sivas, Central Anatolia, traditional Traditional region	Attempted suicide	N = 291 (210 females, 81 males)	2011–2012 Emergency Department of Sivas Numune Hospital	Retrospective evaluation of the hospital records Standardized anonymized documentation forms were filled out by attending staff	**Characteristics** Socio-demographic factors Female gender (72% females vs 28% males) Young age (age group 15–24: 63%) Economic dependence (34% females were housewives) (Mental) Illness History of mental illness (16% females, 24% males) Mental illness (52% females vs. 31% males) **Precipitating Factors** Marital problems (77% females vs. 20% males) Relationship problems with family and a spouse/partner (77%) Domestic violence (18%)
Guloglu, et al. (2009) [47]	Diyarbakir South East Turkey, traditional	Attempted suicide	N = 1281 N = 901 females	2003–2007 Dicle University Hospital	Retrospective evaluation of the hospital records Standardized anonymized documentation forms were filled out by attending staff	**Characteristics** Socio-demographic factors Gender (70% females vs 30% males) Being married (0.55%) Being single (0.43% males only) Higher lethal intent (61% females vs. 39% males)
Ibiloglu et al. (2016) [48]	Diyarbakir South East Turkey, traditional	Attempted suicide	N = 106 N = 57 females N = 49 males Ages: 18–55	Data collection period was not specified Department of Psychiatry at Dicle University Faculty of Medicine	Clinical interviews with the patients admitted to the hospital	**Precipitating Factors** • Relationship problems with their spouse or family (42.5% females)
Konkan et al. (2014) [49]	İstanbul, Western Turkey, less traditional Region	Attempted suicide	N = 102 N = 54 females N = 48 males	2010–2011 Bakırkoy Prof. Dr. Mazhar Osman Mental Health and Neurological Diseases Hospital	Case-control study Self-reported data from patients on their mental health were obtained through validated scales	Precipitating Factors* • Use of “focus on emotions & venting of emotion strategy”, considered to be a non-functional problem-solving strategy (Means: 13.27 among females vs. 11.58 among males)*
Köse et al. (2012) [50]	Van, Eastern Turkey, traditional	Attempted suicide	N = 112 N = 92 females N = 20 males	2009 Van Education & Research Hospital	Retrospective evaluation of the hospital records Standardized anonymized documentation forms were filled out by attending staff	**Characteristics** Socio-demographic factors Female gender (82%) Young age-females aged 15–24 years (71%) Not participating in labour force (being a housewife, 35%) **Precipitating Factors** Family problems (45%) Domestic violence (17%) Loneliness (5.3%)
Mete, Söyiler & Pehlivan (2019) [51]	Turkey, Bingol, Eastern Anatolia, traditional region	Attempted suicide	N = 550 N = 413 (75%) were aged between 16–24	2013–2018 Emergency departments of the hospitals in Bingol	Retrospective evaluation of the hospital records Hospital staff administered monitoring forms of patients treated for attempted suicide	**Characteristics** Socio-demographic factors Female gender (76.5%, 3.2 times more than male gender) Economic dependence (17% females were housewife) **Precipitating Factors** Domestic violence 4.3% (p = 0.01) Family conflicts 21% (p = 0.01)
Ozdel et al. (2009) [52]	Pamukkale, Western Turkey, less traditional	Attempted suicide	N = 144 suicide N = 108 females N = 36 males	2006–2007 Emergency Clinic of Pamukkale University Hospital	Clinical interviews with the patients admitted to the hospital	Characteristics Illiteracy (37% females vs. 19% males) Marital status (36.1% females vs 38.9% males) **Precipitating Factors** Low religious orientation (86.1% females vs 81% males)
Saraçoğlu et al. (2014) [22]	Cukurova, South Turkey, traditional	Attempted suicide	N = 122 N = 84 females N = 38 males	2009–2011 Çukurova University Faculty of Medicine	Clinical interviews with the patients admitted to the hospital	**Characteristics** Socio-demographic factors • Female gender (69%) (Mental) Illness Depression (72% females vs. 27% males) Anxiety (65% females vs. 35% males) Adjustment disorder (61% females vs. 38% males) Bipolar disorder (56% females vs. 45% males) Borderline Personality Disorder (86% females vs. 14% males) Other psychological disorders (67% females vs. 33% males)
Senol et al. (2005) [53]	Erciyes, Central Anatolia, traditional	Attempted suicide	N = 333 N = 209 females N = 124 males	2001–2002 Erciyes University, Faculty of Medicine, Acute and Emergency Department	Mixed methods (clinical interviews with the patients & evaluation of the hospital records)	**Characteristics** Socio-demographic factors Female gender (63%) Young age-females aged 15–24 years of age (66% females vs. 34% males) Being married (48.3%) Being single (69.7% males only) Low education (49%) Not participating in labour force (i.e. being a housewife, 62%) **Precipitating Factors** Domestic violence (50%) Other precipitants related with relationship problems (e.g. getting divorced, not being able to conceive) (63% females vs. 7% males) Family conflicts (74% females vs. 26% males) School failure (65% females vs. 35% males) Job failure (67% females vs. 33% males)
Simsek et al. (2013) [13]	Sanliurfa, South-East Turkey, traditional	Attempted suicide	N = 693 77.6% female 33.4% male	2010 Emergency Service suicide attempt registry record forms from 12 public and private hospitals	Descriptive surveillance study Hospital staff administered monitoring forms of patients treated for attempted suicide.	**Characteristics** Socio-demographic factors • Female gender (77%), female to male ratio (3.47:1) **Precipitating Factors** Family conflict (30% females vs. 21% males) Domestic violence (9% females vs. 4% males) Relationship problems within the family (29.7% females vs. 21% males)
Turhan et al. (2011) [54]	Hatay, South East Turkey, traditional region	Attempted suicide	N = 1613 N = 1270 females N = 343 males	01/2007-12/2009 Emergency services of 8 state hospitals	Retrospective evaluation of the hospital records. Hospital staff administered monitoring forms of patients treated for attempted suicide.	**Characteristics** Socio-demographic factors • Female gender (78,8%) and young age 15–24 (Mental) Illnesses • Mental illness (9% females vs. 13% males) **Precipitating Factors** Relationship problems with opposite gender (14% females vs. 17% males) Domestic violence (14% females vs. 12% males) Economic problems (2% females vs. 8% males) Relationship problems within the family (42% females vs. 33% males) Rape (0,5% females vs. 0% males)
Yasan et al. (2008) [55]	Diyabakir, South-East Turkey, traditional	Attempted suicide	N = 96 subjects who attempted suicide by poisoning for the first time (age 15 and above) 76 cases (51 females, 25 males) were reached at the follow-up after one year.	02/2005-07/2005 University Hospital of Dicle, Diyarbakir 2006: follow-up through a telephone survey	Prospective study Clinical interviews with the patients admitted to the hospital.	**Characteristics** Socio-demographic factors Female gender (69%) Young age: 73% of age group 15–24 were females Low education (76% females had primary education only) Unemployment (87% female) Marital status (27% females vs 33.3% males) (Mental) Illness Depression rate was 51% for both genders, Co morbidity of psychiatric disorders (24% females vs. 6% males) **Precipitating Factors** Religion: both genders were less devoted than the rest of their household. Stressful events within family (e.g. forced into undesirable marriage, discouraged from seeking employment) (62% females vs. 18% males) Follow-up of the precipitating factors Unfavourable attitude of their family (64% females vs. 36% males) Lack of familial or social support (31% females vs.8% males) Family violence after the attempt (65% females vs. 24% males) Persistence of trigger factors (33% females vs. 12% males) Lack of access to therapy (63% females vs. 40% males)
Yektaş et al. (2014) [56]	İzmir, Western Turkey, less traditional	Attempted suicide	N = 79 females aged 15–17 years	Data collection period was not specified Ege University Faculty of Medicine Department of Child and Adolescents Mental Health	Cross-sectional study Self-reported data from patients on their mental health were obtained through validated scales	**Characteristics** [Not assessed] **Precipitating Factors** Precipitating factors for females with no serious intention to die (N = 4): Relationship problems with parents (N = 3.75%) Loneliness (N = 1, 25%) Precipitating factors for females with indefinite intention to die (N = 16) Relationship problems with parents (N = 5, 31.25%) Problems at school (i.e. academic failure) (N = 3, 18.75%) Relationship problems with friends (N = 2, 12.5%) Problems at school & relationship problems with friends (N = 2, 12.5%) Relationship problems with family & friends (N = 4, 25%) Precipitating factors for females with definite/serious intention to die (N = 19) Relationship problems with parents (N = 8, 50%) Relationship problems with friends (N = 2, 12.5%) Relationship problems with family & friends (N = 4, 25%) Conflicts with the authority (e.g. police, school staff) (N = 1, 6.25%) Conflicts with friends, family & authority (N = 1, 6.25%)
Altindag et al. (2005) [28]	Batman, Southeast Turkey, traditional	Suicide	N = 31 (total cases of suicide in Batman in 2000 N = 26 female cases, age 15–24 (with an informant. In 5 cases, no informants were willing to participate). Plus 25 control cases	Batman Public Prosecutor 2000	Controlled psychological autopsy study	**Characteristics** Socio-demographic factors Female gender (female to male ration: 1.7:1)* Young age (two third was between 15–24 years old). Illiteracy, not participating in labour force (i.e. not having paid work)* Precipitating Factors* • Honour related violence (e.g. girls are not sent to school their activities are limited to the home).
Asirdizer et al. (2010) [57]	Inhabitants of the Republic of Turkey	Suicide	N = 22.350 N = 13692 males N = 8658 females	The entire country (Turkey) was covered. Years:1995–2005	Suicide statistics were retrieved from the Turkish Statistical Institutes (TURKSTAT)	**Characteristics** Socio-demographic factors • Economic problems (5.17% females vs 19,0% males) (Mental) Illness • Illness (31.34% females vs 28.42% males) **Precipitating Factors** Relationship problems with the family (i.e. incompatibility with the family) (females: 31.29%, males 16.82%) Relationship problems with a partner/spouse (i.e. failed romantic relationship) (3.82% females vs 2.84% males) Educational Failure (5.46% females, 5.46% males).
Enginyurt et al. (2014) [58]	Inhabitants of the Republic Turkey	Suicide	N = 17342 N = 12107 males N = 5235 females	The entire country (Turkey) was covered. Years 2007–2012	Suicide statistics were retrieved from the Turkish Statistical Institutes (TURKSTAT)	**Characteristics** Socio-demographic factors Being illiterate (7.5% females) Low education (not finished a primary school) (13.9% females vs 7.8% of males) (Mental) Illness • Illness (22.5% vs 18,8% males). **Precipitating Factors** Family problems (14.3% females vs 9.1% males) Relationship problems (i.e. unrequited love) (5.5% females only)
Goren et al. (2004) [59]	Diyabakir, South-East Turkey, traditional	Suicide	N = 302 N = 174 females N = 128 males	Diyarbakir Province Years: 1996–2001	Post-mortem investigation and autopsy reports	**Characteristics** Socio-demographic factors Young age-females below the age of 20 (56.3%) Being married (37% females) **Precipitating Factors** • ‘Deflorated hymen’ (interpretation: honour related issue in traditional family structures) (13% females)
Hekimoglu et al. (2016) [29]	Van, Eastern Turkey, traditional	Suicide	N = 66 (females)	Van City (Turkey) was covered Years: 2005–2011	Post-mortem investigation and autopsy reports	**Characteristics** Socio-demographic factors Young age- 45,45% of the cases was aged between 16–20, and 13.6% of the cases were under the age of 15. Not participating in labour force (i.e. being a housewife/doing household) (87.9%) Economic status was ‘very poor’ for 86.4% of the cases (Mental) Illness • Being under treatment for depression 7.6% **Precipitating Factors** Being married as a fellow wife (4.5%) Being married through bride exchange (15.2%) (one of the nine cases under the age of 15 was married through bride exchange) Physical signs of domestic violence (18.2%)
Karbeyaz et al. (2013) [60]	Eskisehir, Western Anatolia, less traditional	Suicide	N = 553, N = 395 male and 158 were female	The city of Eskisehir, 1997–2011	Post-mortem investigation and autopsy reports	**Characteristics** Socio-demographic factors • Being unemployed (73% females vs 27% males) (Mental) Illness A formal diagnosis of a mental illness (47% females vs 22% males) **Precipitating Factors** Loneliness (35% females vs 23% males) Relationship problems with a partner (27% females vs 17% males) Loss of a relative (21% females vs 11% males)
Karberyaz et al. (2016) [61]	Eskisehir, Western Anatolia, less traditional	Suicide	N = 75 N = 34 females, N = 41 males (75/428 suicide cases were students)	Eskisehir City 2004–2015	Post-mortem investigation and autopsy reports	**Characteristics** (Mental) Illness • Possible diagnosis of a mental illness (64% females)
Oner et al. (2014) [12]	Inhabitants of the Republic Turkey	Suicide	N = 44586 N = 16249 females (36%) N = 28347 males (64%)	The entire country was covered (Turkey)	Post-mortem investigation of annual records of Turkish National Institute of Suicide Statistics	**Characteristics** Socio-demographic factors • Higher proportion of females aged 15–24* **Precipitating Factors** The leading reasons for suicide in females was relationship problems with family and/ spouse* Other reasons (business failure, illness, educational failure) were less common among females compared to males (not broken down by age)*
Oner et al. (2007) [62]	Inhabitants of the Republic Turkey	Suicide	N = 17.327, males: N = 10585, females = 6742	The entire country was covered (Turkey)	Post-mortem investigation of annual records of Turkish National Institute of Suicide Statistics	**Characteristics** Socio-demographic factors Young age-females aged under 15 (z = 8.06, P<0.001, 95% CI.55;90) and between age 15–24 (z = 36.56 P<0.001 CI 2.64: 2.94). Illness (6.90% to 11.52% females in all age groups vs 8.02% to 11,24% males) **Precipitating Factors** Relationship problems (i.e. unsatisfactory relationships) (5.47% to 14.26% females vs 6.15% to 11.48% males). Economic problems (3.39% to 15.99% females vs 5.22% to 14.26% males) Educational Failure (3.31% to 13.25% females vs 6.59 to 11.46% males)
Taktak et al. (2013) [63]	İstanbul, Western Turkey, less traditional Region	Suicide	N = 124, n = 83 male and N = 41 female	Istanbul Forensic Medicine Institute April-August 2002	Post-mortem investigation and autopsy reports	**Characteristics** Socio-demographic factors Young age (mean 29 years, standard deviation 13.8/ males mean age 35.5, standard deviation 17.0)* Being married (44% females vs 49% males) Being unemployed (53.6% females vs 46.4% males). (Mental) Illness A formal diagnosis of a mental illness (32.8% females vs 67.1% males) Under treatment of a mental illness (50% males vs 50% females)

*Percentages were not investigated and/or provided in the paper.

### Characteristics of the included studies

Of all the included studies, 25% (N = 9) were conducted in Europe, 9% (N = 3) in the Netherlands, 6% (N = 2) in Germany, 3% (N = 1) in Austria and 8% (N = 3) in Switzerland. Seventy-five percent (N = 27) were conducted in Turkey. Just over half of all the studies from Turkey (N = 15) were conducted in traditional rural areas, while the remainder (N = 12) were conducted in less traditional, mostly urban areas (see Table 2). Just over half of all studies (62%) from Turkey (N = 17) investigated attempted suicide, while 37% (N = 10) investigated suicide. None of the included studies from Europe investigated suicide.

### Quality assessment of the included studies

More than half of all included studies (62%; N = 21) used data from hospital, crisis centre, public statistics offices or forensic records. There were three (9%) case-control studies and one (3%) prospective study (see Table 1). All but one article [53] collected information either through direct interviews with informants or through the evaluation of available records. Senol and colleagues [53] employed a mixed methods design, including interviews with individuals who attempted suicide and an examination of their hospital records, yet the authors did not list whether discrepancies between these two sources emerged and, if so, how they were addressed.

All the included studies from both Turkey and Europe reported on *characteristics* of suicide and/or attempted suicide. However, *precipitating factors* were reported in 56% (N = 9) of studies on attempted suicide in Turkey, and 60% (N = 6) of studies on suicide in Turkey (see Table 2). *Precipitating factors* were reported in 50% studies (N = 4) from Europe.

### Characteristics of suicide and attempted suicide

#### Socio-demographic factors

*Gender*. Young Turkish women were overrepresented compared to men in studies of attempted suicide in both Europe and Turkey. For instance, among a Turkish speaking patient sample in Vienna, Austria, 71.5% of the suicide attempters were female [40]. The two studies with the most dramatic gender differences relative to attempted suicide (the sex ratio of women to men was 4:1) were conducted in more traditional areas in Turkey [50, 54]. Contrary to these findings, most studies on suicide [12, 57, 58, 60, 61, 63] indicated that men are at more risk than women in Turkey, with one exception [28]: Altindag and colleagues [28] reported a female-to-male suicide ratio of 1.7:1 in a traditional rural area in Turkey.

*Age*. The most frequent age group that presented with a suicide attempt at the hospital was 15–24 year olds in both Turkey and Europe. One of the studies conducted in Switzerland indicated that Turkish women under the age of 25 accounted for the 38.6% of all attempted suicides [41, 42]. Similar findings regarding the high frequency of young women among people who attempted [43, 45, 46, 48, 52, 55] or died by suicide [12, 28, 62, 63] were reported in traditional areas in Turkey. For instance, an article with suicide data derived from the annual reports of Turkey’s suicide statistics (between 1998 and 2000) indicated a high number of suicides among girls under 15 [62].

*Marital status*. Turkish females who attempted suicide were more often married than ethnic-majority women in Europe and Turkish men in Turkey. For instance, 62% of women of Turkish origin in the Netherlands were married compared to 41% of Dutch women [24], and 72% of women of Turkish origin were married compared to 68% of Swiss women [42].

Regarding studies from Turkey, three out of five focused on attempted suicide reported on marital status. These three studies, all conducted in rural traditional areas, showed that being married was approximately 1.5 to 3 times more common for women than men [45, 46, 53]. However, in two studies, marriage frequency was similar among men and women; one was conducted in a traditional area [55] and the other in a less traditional area [52].

*Employment/socio-economic status*. Only one study from Switzerland [41, 42] provided information about the participation of Turkish women in Europe’s labour market, and it reported that not participating in a labour force was three times higher among first-generation Turkish female immigrants compared to Swiss female suicide attempters (30% versus 10%; [41]).

In Turkey, a considerable number of the studies investigating attempted suicide [43, 46, 53, 55] and suicide [28, 29, 60, 63] provided similar findings on labour market participation, indicating that not participating in the labour market was much more common among female suicide attempters and suicide victims than male. All but one of the studies [63] were conducted in traditional areas in Turkey.

Four studies of suicide in Turkey mentioned that a low socio-economic status and poverty was particularly relevant among women [12, 29, 62]. Moreover, one study from a traditional area reported that 86.4% of women aged 16–20 who died by suicide were identified as ‘very poor’ [29]. However, two population-based studies indicated that economic problems were less often reported among women than men who died by suicide (57% versus 19%; [12, 57]).

*Education*. Only one European study from Germany assessed educational levels and reported that 20% of women from the second generation aged 18–34 had obtained a higher level of education, while none of from the first generation aged 35–49 and/or 50 and above had acquired a higher education [25]. However, the study’s authors noted that these educational differences reflect the general Turkish immigrant population.

Three studies conducted in Turkey investigating suicide [28, 58] and one study investigating attempted suicide [50, 52] presented findings of illiteracy stratified by gender. These studies found that illiteracy was two to three times higher among women than men, which reflects the gender gap in illiteracy in the general Turkish population. Three studies [28, 50, 52] reported high percentages of illiterate women. For instance, study 28 reported 42% of female victims to be illiterate (women in the general population: 7.5%). However, these high percentages of female illiteracy seem influenced by the area/site of the studies (tradition, rural areas).

*Religiosity*. Only one study from Germany systematically investigated religion and showed that all Turkish female immigrants who attempted suicide identified as Muslim [25], but the study did not assess the strength of their religious identification. However, two studies from traditional areas in Turkey reported high rates of low religious devotion among Turkish female suicide attempters compared to males [52, 55]. Religiosity was not assessed in any of the studies investigating suicide in Turkey.

#### Mental illness and adjustmentor coping

There was no clear pattern regarding the comparative relevance of depression among Turkish women in Europe. For instance, one study from the Netherlands [24] showed that ‘mood disorders’ were approximately 1.5 times more common in Dutch female suicide attempters compared to Turkish female immigrants (59% versus 38%). However, another study from the Netherlands [39] showed that depression was more common in Turkish female immigrants than in Dutch women (no percentages provided). The latter finding aligns with a Swiss study [41] that showed that Turkish women were approximately 1.5 times more likely to report an ‘affective disorder’ than Swiss ethnic-majority women (51% versus 31%).

In Turkey, two of five studies found that depression was more common among women than men who attempted suicide [22, 44]. For instance, one study from a traditional area reported that depression was three times more likely among female attempters than males (72% versus 27%; [22]). One of the five studies did not investigate gender differences in mental illness [48].

‘Adjustment’ and ‘stress-related disorders’ were frequently reported among Turkish female suicide attempters in Germany (49.7%) [23] and Switzerland (45.7%) [38]. In the Swiss study [38], a comparison showed that adjustment disorders were less often reported among ethnic-majority women (14.6%) than Turkish immigrant women. Additionally, a study in Turkey noted large gender differences in adjustment disorder (61% versus 38%) among female attempters compared to males [22]. Regarding suicide cases in Turkey, three studies reported that mental illness was more common among female suicide attempters than males, yet these studies did not specify the type of mental illness [60, 61, 63]. Finally, the use of emotion focused coping was found somewhat more often among Turkish females than males who attempted suicide (Means: 13.27 among females vs. 11.58 among males [49]).

#### Precipitating factors related to suicide and attempted suicide

*Relationship problems with spouses or families*. Five of seven studies on attempted suicide from Europe [23–25, 40–42] and eight of 11 studies from Turkey [13, 44, 45, 50, 51, 53, 54, 56] highlighted relationship problems with spouses or families as precipitating factors. For example, one study conducted in Berlin found that 61% of female attempters reported a prior conflict with their spouse as the reason for the attempt [23]. Another study from Berlin illustrated how family problems–mostly related to a hierarchical family structure and the pressure to put the family first (i.e., the self-sacrifice of women)–triggered the suicidal behaviour [25]. Similar aspects were reported regarding attempted suicide in Turkey. For instance, Cetin and colleagues reported that females from Istanbul who had attempted suicide perceived their role in their family as negative and felt this way significantly more often than young female controls who had not attempted suicide [44]. Similarly, ‘incompatibility with the family’ was reported among 31% female suicide cases compared to 17% of male cases in Turkey [57]. Six out of eight studies from rural areas in Turkey pointed to the relevance of the marital relationship and family problems to suicidal behaviours in women [13, 45, 50, 51, 54].

*Domestic violence*. Domestic violence perpetrated by a spouse or family member was frequently reported in studies investigating attempted suicide in Europe and Turkey. One study identified domestic violence as the main precipitating factor for attempted suicide among Turkish females in Switzerland (21% first generation; 15% second generation), whereas none of the Swiss women reported domestic violence as the reason for their attempt [41]. Additionally, domestic violence was listed as an important precipitant for attempted suicide among women in eight studies that were conducted in traditional areas in Turkey [13, 43, 45, 46, 51, 53–55]. Frequencies varied from 4–9% [45, 51] to 50% [53]. In terms of gender differences, domestic violence was twice (9% women versus 4% men) [13] and four times (9% women versus 2% men) [45] more common among women than males in traditional areas in Turkey. Only one study from a traditional area indicated that the gender differences in violence were relatively small (12% men versus 14% women; [54]). Furthermore, in the studies investigating suicide, domestic violence was mentioned once. Hekimoglu and colleagues concluded that physical violence possibly played a role in one fifth of all female suicide cases in their sample based on the physical signs of trauma that were observed during post-mortems [29].

Sexual abuse was infrequently mentioned as a precipitating factor. One exception was a study from the Netherlands that indicated it was less often reported as a precipitating factor among Turkish women compared to Dutch women who attempted suicide (19% versus 38%) [24]. Furthermore, only one study in Turkey on attempted suicide listed ‘rape’ as a precipitating factor and reported small percentages (0.5% females versus 0% males) [54]. Sexual abuse was not reported as a precipitant among the studies investigating suicide.

*Honour-related violence and personal autonomy restrictions*. In Europe, van Bergen and colleagues indicated that 55% Turkish of women reported honour-related conflict (e.g., being accused of not maintaining sexual abstinence until marriage) as a precipitating factor in their attempted suicides [24]. A study in Germany reported that conflicts between Turkish women and their parents and/or spouses were linked to family honour [25]. Moreover, honour-related issues were mentioned as precipitating factors in the discussion section of nine of the 16 studies investigating attempted suicide in Turkey [11, 13, 43, 47, 50, 51, 53, 55]. All but one of these studies [11] were conducted in traditional regions of Turkey.

One study from Berlin concluded that collectivism (family centeredness) in relation to family honour led to the infringement of women’s personal autonomy [25]. A study based in Amsterdam reported family accusations against women who violated ‘chastity’ codes, resulting in threats from the family and additional social repercussions [24]. One study investigating attempted suicide in a traditional area in Turkey explicitly reported autonomy restrictions as a precipitating factor [55]. Specifically, the authors reported that 11% of women were forced to maintain an undesirable marriage and that 11% were discouraged from seeking employment, while 27% reported ‘interference with their social life’ [55].

Two studies from traditional regions provided evidence of autonomy restrictions as a potential trigger for suicide [29, 59]. One study reported that 15.2% of women aged 16 and 20 and just over 10% of women under the age of 16 were married through ‘bride exchange[s]’ [29]. The other study reported that ‘not maintaining sexual abstinence until marriage’ was listed as a reason for conflict with the family among 13% of female suicide cases [59].

*Migration-related factors*. Two precipitating factors seemed to be related to migration: culture conflict and discrimination. Yet, these factors were only reported in one study from Germany [25]. Turkish women in Berlin often felt they could not meet the socio-cultural expectations of German society, such as being strong, independent and assertive, because they felt that Turkish culture demanded the opposite: that they be submissive and family oriented. Furthermore, Turkish women reported that they perceived discrimination against them and a lack of acceptance from German society as leading to feelings of social exclusion and isolation. Furthermore, women who were recent immigrants mentioned a lack of social support (being away from their natal family) and language difficulties as important acculturation-related stressors.

## Discussion

The results of this systematic review highlight rather similar characteristics and precipitating factors in all the included studies, regardless of the country/continent (Turkey versus Europe) or the type of suicidal behaviour (attempted suicide or suicide). Our analyses of sociodemographic factors showed that Turkish women who demonstrated suicidal behaviours were relatively young and married at a relatively young age. They were often not part of the labour force, and tended to have low socio-economic status. Another remarkable finding was that some studies indicated that religiosity among women who attempted suicide in traditional areas of Turkey was very low [52, 55]. This may suggest that women who are not religious could experience a sense of isolation because they are surrounded by mostly strict believers in traditional areas. The latter argument is supported by recent studies showing that in Muslim countries where the freedom of religion is restricted or religion is compulsory, the protective function of religion could be limited [2, 65].

Our analysis of the role of mental illnesses showed that women often presented symptoms of mental illnesses, such as depression. Moreover, in some studies, mental illness (notably depression) was more commonly reported among women than men or ethnic-majority women (in Europe). However, it is needed to delve deeper into what would cause depressive symptoms in women. This relates to the work of Devries and colleagues, who provided meta-analytical evidence of the predictive role of intimate partner violence on symptoms of depression and attempted suicide among women worldwide [66]. Arguably, the frequency of mental disorders in this current review points to a lack of physical, sexual and emotional safety among women. Impaired autonomy, economic dependence and poverty render women particularly vulnerable to distress, which is a tremendous setback to mental health equity [30, 67]. An interesting finding is that women living in traditional areas of Turkey reported less mental illness than those living in less traditional areas. However, the underreporting of mental illness due to stigma in traditional areas may explain this result [68]. Alternatively, the lack of mental health professionals in traditional regions who could provide a diagnosis could also explain it. On the other hand, psychiatric diagnoses of individuals who died by or attempted suicide may reflect a tendency of scientists to medicalise social and political problems, including violence, while the problem of high suicidality should be combatted as a form of social oppression [69, 70].

As for precipitating factors of suicidal behaviours, it was striking that women in the studies had often been victims of violence and family conflicts. The cross-national consistency of the characteristics and precipitants of suicidal behaviours among young Turkish women in Europe and Turkey provides further support for the continuous influence of socio-demographic affronts and violence- and interpersonal-conflict-related factors that exist in pre- and post-migration contexts. Furthermore, there is evidence indicating that violence against women may be labelled as ‘relationship problems’ or ‘family conflict’ [24, 25]. These violations are gender specific and more often a cause for concern for women than men. Cross-culturally, there is also well-documented evidence highlighting that violence against women from their family or spouse is crucial to suicidal behaviours, especially among women of ethnic minority or immigrant backgrounds [67, 71] and that this type of violence triggers suicidal behaviours [67].

One of the important highlights of the current study is the evidence of the restrictions on personal autonomy in the lives of Turkish women in both continents. Women and girls who attempt or die by suicide, particularly if they live in or originate from rural areas of Turkey, seem to be confronted with situations in which their input on strategic choices regarding marriage, divorce, labour market participation and sexuality is infringed upon or denied. Rural areas of Turkey are well known for their practice of conservative gender typing and patriarchal family structures [8, 9, 27, 28]. Although honour-related violence and autonomy restrictions were not always investigated as precipitating factors (28% studies from Europe and 12% from Turkey examined it), it is suspected that the within the ‘domestic violence’ and ‘family problems’ categories, honour played a role as well. A study (not part of our review) of a large community sample of women aged 13–54 (N = 414) in Turkey provided extensive evidence of honour-related violence in traditional rural areas [72]. Accordingly, 55.8% of women under 20 included in this report were married through an arranged marriage, and 24.5% were forced into a marriage. Furthermore, all types of violence were frequently mentioned by these women (99.3% psychological violence, 89.4% physical violence, 91.3% verbal violence, 90.8% economic violence and 63% sexual violence) [72].

Comprehensively viewed, the results of our review could be interpreted within the framework of intersectionality in which the broader structural inequalities in gender roles and expectations, such as self-sacrifice for the sake of honour, and power imbalances among social classes, such as the low socio-economic status of women as well as poverty, collectively impact the suicidal behaviours of Turkish women [2, 5, 25, 29, 55, 69, 73]. Moreover, the consistency of socio-demographic characteristics emphasises that young Turkish women often occupy the lowest social status in highly traditional Turkish communities compared to males and older women and men [10, 72, 74].

Furthermore, the precipitants related to autonomy restrictions also confirm features of Durkheim’s fatalistic suicide model, which suggests that suicide is the result of a lack of control over one’s life, which seems to be dictated by powerful others [31]. In a Durkheimian sense, a fatalistic suicide communicates a sense of alienation in response to harsh moral demands upheld through force [25, 32, 75]. Additionally, drawing on the interpersonal theory of suicide developed by Joiner [76], it could be argued that suicide attempts and suicides are strategies to escape the sense of powerlessness and abuse that evoke feelings of thwarted belongingness [77].

### Strengths and limitations

An important asset of this study is that it is comprehensive in the sense that it covers both suicide and attempted suicide, and it reports on the quality of the included studies. Another strength of this review is that it draws on the literature published in four languages (Turkish, Dutch, German and English) and synthesises knowledge on the suicidality of Turkish women from Turkish and European contexts.

Nevertheless, this study also has limitations. We were unable to present results about ethnic differences, as the studies we included in the review did not specify the (sub)ethnic background of participants. This is unfortunate as Turkey is host to people with a variety of ethnic backgrounds (Kurds, Circassian, Arabs Turks and so on), and these ethnicities may convey various links to suicidal behaviour. Next, with respect to the quality of the studies, some did not consistently present gender differences, while others fell short of in-depth examinations. Furthermore, several studies relied on a single source of information, such as hospital records, and some studies only examined a small number of factors. In addition, since the majority of studies of attempted suicide were conducted very shortly after women were admitted as patients to a hospital, reports by hospital staff were much more common than self-report. Another issue was that some studies that applied a mixed method or multiple informant approach did not describe how they dealt with potential discrepancies between different sources. These flaws or omissions in methodology or the presentation of data indicate that the conclusions of some of the studies may be biased. Another limitation is that the reviewed studies rarely used control groups. Without a comparison group, the uniqueness of certain characteristics and/or precipitants remains unclear. Additionally, prospective studies investigating interpersonal relations and circumstances following an attempt, or repetition of an attempt, were scarce. Such a paucity prevents a solid check for support or care for women after an attempt or the monitoring of possible variations in precipitating factors within cases over time.

Another limitation of this study is the possible variability of its data collection, reporting and selection of the analysed factors across countries and Turkish regions. As briefly mentioned in the beginning of our discussion, honour-related violence was not sufficiently empirically investigated in the studies from Turkey, and only one study from Europe assessed discrimination and stigma against immigrants. This means that the role of migration and belonging to an ethnic immigrant minority group in Europe for understanding suicidal behaviour of Turkish women remains inconclusive. Finally, as this literature review was narrative in fashion, we have not tested outcomes across studies through statistical measures.

## Conclusions

To our knowledge, this is the first systematic literature review comparing the characteristics and precipitating factors associated with suicidal behaviours in Turkish females in Europe and Turkey, and its results point to a consistency between the two geographies. Future research should systematically address gender differences in the investigation of precipitating factors and characteristics, include a representative control group and make more use of prospective designs. Furthermore, future studies could investigate whether the risk and precipitating factors of suicide among women are influenced by traditional culture and patriarchal systems, and they could explicitly examine the harmful role of honour. For the latter, qualitative or mixed methods designs may be appropriate.

In summary, the suicidal behaviours of Turkish women are related to a complex interaction of cultural, social, economic and individual factors that vary depending on region and socio-political contexts [2]. Suicidality among Turkish women can be interpreted as an attempt to communicate their discontent in response to social injustices and/or an attempt to escape from these injustices, which they are exposed to in the name of honour [25]. These are clear examples of serious human rights violations [2]. There is empirical evidence from the Netherlands indicating that change of cultural beliefs in gender-specific honour codes is possible through psycho-educational training programmes involving ethnic groups that place considerable value on honour [10]. In addition to such programmes, culturally and gender-sensitive services for survivors of domestic violence and early intervention and/or detection of young women at risk are important. See, for example, the awareness campaign End Your Silence Not Your Life, which targets Turkish immigrant women in Berlin [10]. Finally, adequate jurisdictional and political action to prevent human rights violations against women in Europe and Turkey are much needed in order to prevent suicidal behaviours [78, 79].

## Supporting information

S1 Checklist PRISMA 2009 checklist. (DOCX)
